# Simple-Random-Sampling-Based Multiclass Text Classification Algorithm

**DOI:** 10.1155/2014/517498

**Published:** 2014-03-19

**Authors:** Wuying Liu, Lin Wang, Mianzhu Yi

**Affiliations:** ^1^Department of Language Engineering, PLA University of Foreign Languages, Luoyang, Henan 471003, China; ^2^College of Computer, National University of Defense Technology, Changsha, Hunan 410073, China; ^3^College of Humanities and Social Sciences, National University of Defense Technology, Changsha, Hunan 410073, China

## Abstract

Multiclass text classification (MTC) is a challenging issue and the corresponding MTC algorithms can be used in many applications. The space-time overhead of the algorithms must be concerned about the era of big data. Through the investigation of the token frequency distribution in a Chinese web document collection, this paper reexamines the power law and proposes a simple-random-sampling-based MTC (SRSMTC) algorithm. Supported by a token level memory to store labeled documents, the SRSMTC algorithm uses a text retrieval approach to solve text classification problems. The experimental results on the TanCorp data set show that SRSMTC algorithm can achieve the state-of-the-art performance at greatly reduced space-time requirements.

## 1. Introduction

Multiclass text classification (MTC), a supervised learning task of classifying text documents with predefined multiple categories, has been widely investigated since the early days of artificial intelligence [1]. In the current era of big data [2], people pay more attention to the space-time overhead of MTC algorithms to guarantee good effectiveness. Token frequency is a very effective feature in MTC algorithms. If we only use token frequency features in a closed text collections, the feature with one occurrence will never be used, we can easily remove these useless long tail features for lower space-time costs. But in an actual situation, we meet a puzzle of open feature space. The power law of word frequency in a set of text documents was discovered for a long time [3], which may bring an opportunity to propose a novel MTC algorithm.

In this paper, we reexamine the power law of token frequency in a Chinese web document collection. According to the investigation of potential useless features in the closed text collections, we propose a simple-random-sampling-based MTC (SRSMTC) algorithm. The SRSMTC algorithm makes use of a token level memory to store the labeled documents and only uses relative frequency features among tokens. Labelled text documents trigger a memory-based learning, which uses a simple random sampling (SRS) method to reduce the space complexity. Unlabeled text documents trigger a memory-based predicting, which uses an ensemble Bayesian method to reduce the time complexity.

The rest of this paper is organized as follows. In Section 2, we describe some related works. In Section 3, we investigate the power law of token frequency and analyze the potential uselessness rate. In Section 4, we propose the SRSMTC algorithm, in which we use an index data structure to store token level labeled examples. In Section 5, the experiment and result are described. At last, in Section 6, the conclusion and further work are given.

## 2. Related Work

Recently web document classifying is normally defined as a batch MTC problem, which can be simulated as a 12-category web document classifying task [4]. Many batch MTC algorithms have been introduced to deal with the web document classifying. For instance, (1) the* k*-nearest neighbor (kNN) TC algorithm [5] decides a document according to the* k* nearest neighbor document categories; (2) the centroid TC algorithm [6] is based on the assumption that a given document should be assigned a particular category if the similarity of this document to the centroid of its true category is the largest; and (3) the winnow algorithm [7] uses a multiplicative weight-update scheme that allows it to perform much better when many dimensions are irrelevant. Except the above algorithms, the structured feature of documents and the token frequency distribution feature of documents are both crucial to the classification performance. Previous research shows that the structured feature of documents supports the divide-and-conquer strategy and can be used to improve the classification performance [8]. Previous research also shows that the token frequency distribution follows the power law [9], which is a prevalent random phenomenon in many text documents.

The previous algorithms pursue the high classification accuracy and the high overall performance of supervised learning, without more claiming the low space-time complexity. However, the algorithm is space-time-cost-sensitive for many real-world big data applications. Especially, it is unreasonable to require an industrial MTC algorithm with a time-consuming training or updating, such a requirement defeats previous complex statistical algorithms and motivates us to explore a space-time-efficient MTC algorithm.

## 3. Reexamination of Power Law

It has been found that the frequency distribution of words follows the power law within most text documents. In order to validate that the power law also exists in Chinese web documents, we calculate the number of each token in the documents.

### 3.1. Corpus

The Chinese web documents corpus is the TanCorp (http://www.searchforum.org.cn/tansongbo/corpus.htm) collection, which contains a total of 14,150 documents and is organized in two hierarchies. The first hierarchy contains 12 big categories and the second hierarchy consists of 60 small classes. This corpus can serve as three TC datasets: one hierarchical dataset (TanCorpHier) and two flat datasets (TanCorp-12 and TanCorp-60). In this paper, we use TanCorp-12.

### 3.2. Token Frequency Distribution

According to the widely used vector space model (VSM), a text document is normally represented as a feature vector, and each feature is a text token. Previous research has shown that overlapping word-level* k*-grams token model can achieve promising results [10]. But different languages have different appropriate* k* values, and these different representational granularities determine the total number of text features. Here, we consider four overlapping word-level* k*-grams token models (1-grams, 2-grams, 3-grams, and 4-grams) to represent tokens.

**Figure 1:**
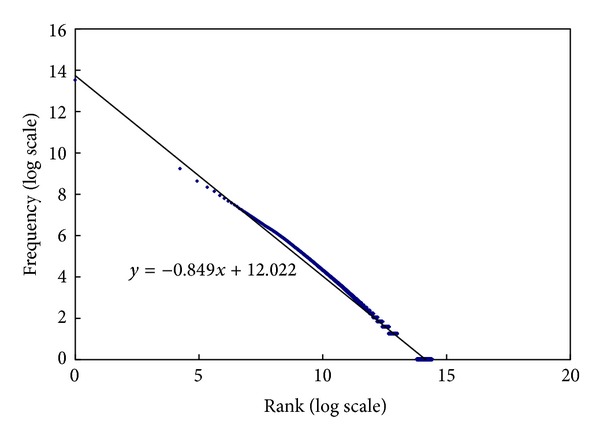
Token frequency as the function of the token's rank with the word-level 2-grams token model in the TanCorp collection.

We calculate the number of each token occurrence in the TanCorp collection.  Figure 1 shows the token frequency as the function of the token's rank with the word-level 2-grams token model. The trendline shows a power law distribution.

The statistical results show that not only the 2-grams token frequency distribution in the TanCorp collection follows the power law, but the others* k*-grams token frequency distribution also follows the power law.  Table 1 shows the detailed trendline coefficients **a** and **b**.

The above reexamination shows that the token frequency distribution follows the power law in the Chinese web documents. The ubiquitous power law indicates that the weightiness of each token feature is not equivalent, which suggests a feature selection method to remove those useless features for lower space-time costs.

### 3.3. Potential Useless Feature

In statistical MTC algorithms, the text feature selection is a widely used method against the high dimensional problem and has a crucial influence on the classification performance. The iteration, the cross-validation, and the multipass scans are all effective methods to the text feature selection. But these methods bring the high space-time complexity. If we can detect and remove those useless features, we will save more time and space. However, what is the useless feature and how to find it?

In a whole text documents set, a token feature with a less frequency (≤2) is a potential useless feature. As an extreme instance, if a token feature occurs only once in a closed training set, it is useless because it will never be used in the training set. So the useful features will not decrease after removing the useless features. We further define the uselessness rate *R*_*u*_ as the ratio of the number of token features with less frequency to the total number of token features. Here, we only consider the word-level 2-grams token in the TanCorp collection.  Table 2 shows the feature number and related uselessness rate in the collection.

The *N*(1) and *N*(2) separately denote the number of token features which only occurs once and twice in the documents set. The *N*(∗) denotes the total number of token features. The *R*_*u*_(≤1) and *R*_*u*_(≤2) are defined as (1) and (2) separately. Consider the following:

(1) $$\begin{array}{c} R_{u}\left(\leq 1\right)=\frac{N\left(1\right)}{N\left(*\right)}, \end{array}$$

(2) $$\begin{array}{c} R_{u}\left(\leq 2\right)=\frac{N\left(1\right)+N\left(2\right)}{N\left(*\right)}. \end{array}$$

Table 2 shows that the uselessness rate is between 63% and 79% in the TanCorp collection. If we can get the whole text documents before predicting, we will easily find these useless token features and cut this long tail. However, the actual application faces an open text space problem, and we cannot foreknow a token feature's occurrence in the open testing. Though an open text space makes it impossible to find these a posteriori useless token features, the higher uselessness rate indicates that there are lots of useless token features. Supported by the a priori and ubiquitous power law, this paper proposes a SRS method to remove these useless token features at the time of training. The range of uselessness rate indicates the theoretical tolerant range of training feature loss rate.

## 4. SRS-Based MTC Algorithm

Previous VSM representation faces an open text feature space and cannot foreknow the vector space dimension. This section presents a data structure of token level memory (TLM), based on which the SRSMTC algorithm smoothes the open text space problem and is space-time-efficient owing to the TLM data structure.

### 4.1. Token Level Memory

In this paper, the object categories of the MTC problem are represented as a set in the form (**C** = {*C*_*i*_}, *i* = 1, 2,…, *n*), and a document **D** is represented as a sequence of tokens in the form (**D** = *T*_1_ *T*_2_ … *T*_*j*_…). Here, we use the overlapping word-level* k*-grams model to define a token. The token frequency within training labeled documents, the key feature of supervised machine learning, implies rich classification information and must be stored effectively [11]. The TLM is a data structure to store the token frequency information of labeled documents, from which we can conveniently calculate the Bayesian conditional probability **P**(*C*_*i*_ | *T*_*j*_) for the object category *C*_*i*_ and the token *T*_*j*_. We straightforwardly combine the Bayesian conditional probabilities of tokens and choose the category of the biggest probability as the document's final category prediction.

**Figure 2:**
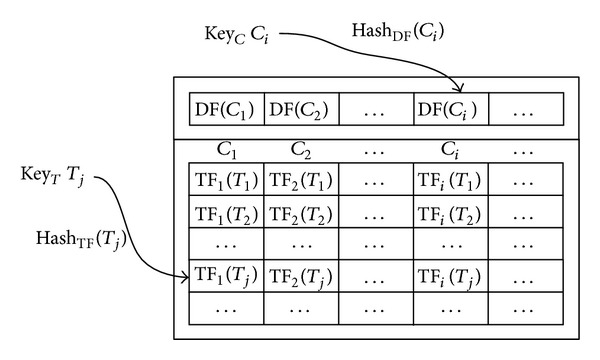
Token level memory.

Figure 2 shows the TLM structure, including two indexes organized as two hash tables. The table entry of the DF index is a key-value pair 〈key_*C*_, value〉, where each key *C*_*i*_ denotes the *i*th category and each value DF(*C*_*i*_) denotes the total number of documents with *C*_*i*_ category labels. The hash function hash_DF_(*C*_*i*_) maps the category *C*_*i*_ to the address of the DF(*C*_*i*_). The table entry of the TF index is also a key-value pair 〈key_*T*_, value〉, where each key *T*_*j*_ denotes a token and each value consists of *n* integers. The integer TF_*i*_(*T*_*j*_) denotes the occurrence times of the token *T*_*j*_ in labeled *C*_*i*_ category documents. The hash function hash_TF_(*T*_*j*_) maps the token *T*_*j*_ to the address of the *n* integers.

The TLM stores labeled tokens, the tiny granularity labeled examples, while other memory-based algorithms, such as kNN [12], store document-level labeled examples. On the one hand, this index structure has a native compressible property of raw texts. Each updating or retrieving of index has a constant time complexity. On the other hand, the power law distribution can help us to remove lots of long tail tokens.

### 4.2. Pseudocode of SRSMTC Algorithm

Supported by the TLM, the SRSMTC algorithm takes the supervised training process as an updating process of indexes and also takes the predicting process as a retrieving process of indexes.

According to the power law, we add a SRS into the supervised training process. The SRS idea is based on the assumption that some tokens selected randomly according to equiprobability trend to be higher frequency tokens. If only the relative frequency features are concerned among tokens, we can use partial tokens of a labeled document to update the TLM after SRS. As a result, lots of long tail tokens will be removed, and the relative frequency will not change among tokens. We define the SRS rate *R*_srs_ as the ratio of the number of tokens added into the TLM to the total number of tokens of each labeled document, which is a real number (*R*_srs_ ∈ [0, 1]).

**Figure 3:**
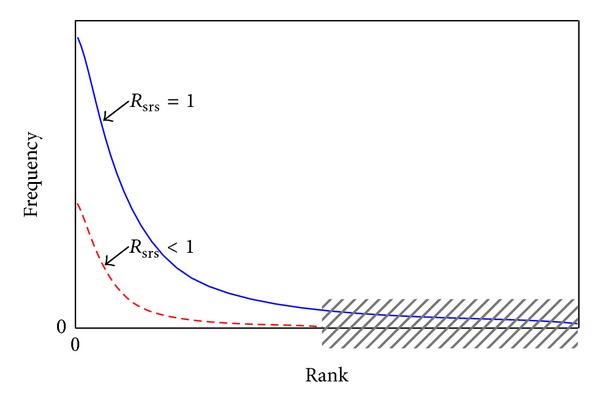
SRS sketch.

Figure 3 shows the SRS sketch. The horizontal-axis (*x*-axis) indicates the token's rank, and the vertical-axis (*y*-axis) indicates the token frequency. If the SRS rate is one, all the tokens of a labeled document will be added into the TLM at the training time. While if the SRS rate is less than one, there will be some tokens absent in the TLM. Along the updating, these above two cases will form two power law curves in Figure 3, where the shadow range denotes removed tokens. These two power law curves also indicate that the SRS will not change the total distribution of the relative frequency among tokens. Of course, if the SRS rate approximates zero, the classification ability of the TLM will also be damaged. However, what is the optimal SRS rate? Theoretically, a promising SRS rate is the (*R*_srs_ = 1 − *R*_*u*_). But the exact *R*_*u*_ is also not a priori value. Fortunately, the ubiquitous power law gives an approximate heuristic, such as the 20 | 80 rule of the *R*_srs_ | *R*_*u*_.

Algorithm 1 gives the pseudocode of SRSMTC algorithm. When labeled documents arrive, the training procedure only needs to add each document's tokens into the TLM. This procedure firstly analyzes each document text and extracts tokens based on an overlapping word-level* k*-grams model, and then samples the tokens based on a preset SRS rate, and finally updates the token frequency or adds a new index entry to the TLM according to the tokens after the SRS.

The Bayesian conditional probability predicting is a very classical method. According to each observed token of a document, the Bayesian method can obtain an array of probabilities, reflecting the likelihood that the classified document belongs to each category. The ensemble method uses arithmetical average to combine the multiarray of probabilities predicting from all tokens to form a final array. And then, the category of the maximal probability in the final array is predicted as the document's category. When testing documents arrive, the predicting procedure is triggered: (1) the procedure also analyzes each document text and extracts tokens based on an overlapping word-level* k*-grams model; (2) the procedure retrieves the current TLM and calculates each token's probabilities array according to the Bayesian conditional probability; (3) the procedure assumes that each token's contribution is equivalent to the final probabilities array and uses the arithmetical average method to calculate a final ensemble probabilities array; and (4) the procedure chooses the maximal probability in the final ensemble probabilities array and outputs each document's category predication.

### 4.3. Space-Time Complexity

The SRSMTC algorithm mainly makes up of the training and the predicting procedures, whose space-time complexity depends on the TLM storage space and the loops in the two procedures.

The TLM storage space is efficient owing to two reasons: the native compressible property of index files [13] and the SRS-based compressible property at the time of updating. Hash list structure, prevailingly employed in information retrieval, has a lower compression ratio of raw texts. Though the training documents may be large-scale, the TLM storage space will only increase slowly. The native compressible property of index files ensures that the TLM storage space is theoretically proportional to the total number of tokens and not limited to the total number of training documents. The SRS-based compressible property of TLM is caused by the power law of token frequency distribution and the only requirement of relative frequency. The SRS-based feature selection can cut the long tail useless features. The above two compressible properties make that the labeled documents can be space-efficiently stored.

The updating or retrieving of TLM has a constant time complexity according to hash functions. The training procedure is lazy, requiring no retraining when a new labeled document added. Algorithm 1 shows that the time cost per updating is only proportional to the total number of tokens in each document. There are no time-consuming operations. The major time cost of the predicting procedure is related to the number of categories. The straightforward calculating makes that the time complexity is acceptable in actual applications.

## 5. Experiment

We conduct a web document classifier (*wdc*) generated by the proposed SRSMTC algorithm, run the* wdc* classifier on the 12-category Chinese web document classifying task, and compare the results of the* wdc* classifier as well as to that of the kNN classifier, the* centroid* classifier, and the* winnow* classifier. In this experiment, we use the TanCorp-12 corpus and the classical performance measures (MicroF1, MacroF1). The hardware environment for running experiments is a PC with 1 GB memory and 2.80 GHz Pentium D CPU.

### 5.1. Implementation and Evaluation

The* wdc* classifier is an implementation of the SRSMTC algorithm with 12 categories in Chinese texts. In order to extract word-level tokens, we implement a Chinese segmenter in the* wdc* classifier. In the experiments, we do nothing about text preprocessing, such as stemming and stop word elimination.

The web document classifying task is a 12-category Chinese web document classifying task. We use threefold cross-validation in the experiments by evenly splitting the TanCorp-12 dataset into three parts and use two parts for training and the remaining third for testing. We perform the training-testing procedure three times and use the average of the three performances as the final result. Here reports MacroF1 and MicroF1 measures [14], which combine recall and precision over the different categories. The MacroF1 and the MicroF1 emphasize the classification performance of a classifier on rare and common categories, respectively.

### 5.2. Results and Discussions

The experiment includes two parts, the one evaluates the effectiveness of the SRSMTC algorithm in the batch MTC application; and the other verifies the SRS-based compressible property of the TLM data structure.

In the first part experiment, the* wdc* classifier runs on the 12-category Chinese web document classifying task and sets its SRS rate *R*_srs_ = 1. Through evenly splitting the TanCorp-12 dataset, we make the threefold cross-validation. The mean MacroF1 and the mean MicroF1 are shown in Table 3. In Table 3, the results of other four classifiers are cited from existing researches [4]. The results show that the* wdc* classifier can complete classifying task in high MacroF1 (0.8696) and high MicroF1 (0.9126), whose performance exceeds the* centroid*'s, the kNN's, the* winnow*'s, and approaches to the best* SVM* classifier's MacroF1 (0.9172) and MicroF1 (0.9483).

In the second part experiment, we also run the* wdc* classifier under different SRS rate *R*_srs_ from the 90% down to the 10%. The* wdc* classifier repeatedly runs 30 times for each SRS rate, and here reports the mean performance among the 30 results for each SRS rate.

The detailed SRS rate (*R*_srs_), training indexed token compressing rate (*R*_tc_), and performances are shown in Table 4. Where the *R*_tc_ is a posteriori value after the training and is defined as the ratio of the token number in the TLM after the training to the total number of processed tokens during the training. On average of the 30 results, Table 4 shows that the training indexed token compressing rate approximates a direct ratio of the SRS rate, which proves that SRS-based token feature selection according to the theoretical uselessness rate heuristic between 63% and 79% is effective.

## 6. Conclusions

This paper investigates the power law distribution and proposes a SRSMTC algorithm for the MTC problem. This algorithm is an excellent industrial algorithm for its easy implementation and convenient transfer to many applications. Especially, the SRSMTC algorithm can achieve the state-of-the-art performance at greatly reduced space-time complexity, which can satisfy the restriction of limited space and time for many actual big data applications.

The experimental results show that the SRSMTC algorithm can obtain a comparable performance compared with other advanced machine learning MTC algorithms in Chinese web document classifying application. Based on the research of this paper, we can draw following conclusions.

- The token frequency distribution follows the power law, which is a prevalent random phenomenon at least in Chinese web documents. According to the power law, we find many potential useless features and succeed in SRS-based feature selection.
- The token level memory uses an index data structure to store labeled documents. This structure has a native compressible property of raw texts. Each updating or retrieving of index has a constant time complexity, which can satisfy the space-limited and real-time requirements.
- The text retrieval approach can solve the text classification problem supported by the token level memory. Using linear combination ensemble of Bayesian conditional probabilities predicted from token features, the straightforward occurrence counting of token features can obtain promising classification performance. This straightforward counting also brings time reducing.

A web document may belong to the hierarchical category or may have multiple category labels. Further research will concern the multihierarchy MTC problem and the multilabel MTC problem. According to the ubiquitous power law, the SRSMTC algorithm is more general and can be easily transferred to other MTC applications.

## Figures and Tables

**Algorithm 1:**
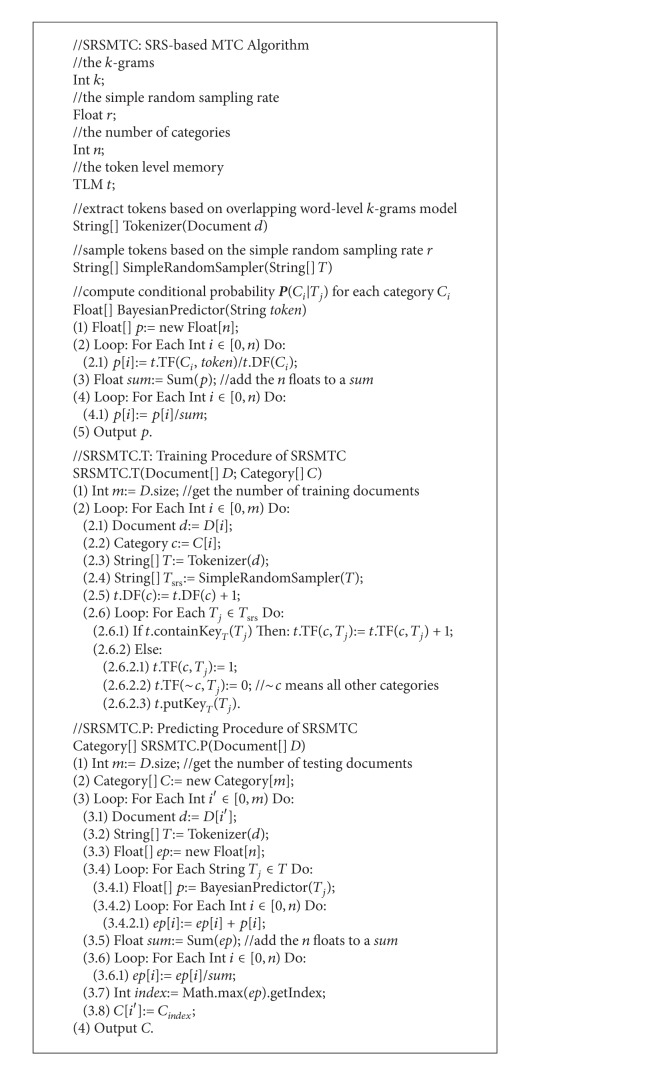
Pseudo-Code of SRSMTC Algorithm.

**Table 1:** Trendline (*y* = **a** *x*  +  **b**) coefficients in TanCorp collection.

	1-grams	2-grams	3-grams	4-grams
**a**	−1.766	−0.849	−0.460	−0.280
**b**	20.129	12.022	6.879	4.242

**Table 2:** Feature number and uselessness rate in TanCorp collection.

Feature number (num)			*R*_*u*_ (%)	
*N*(1)	*N*(2)	*N*(∗)	*R*_*u*_(≤1)	*R*_*u*_(≤2)
1,316,422	325,834	2,087,815	63	79

**Table 3:** MacroF1 and MicroF1 results.

	MacroF1	MicroF1
*SVM *	0.9172	0.9483
*wdc *	0.8696	0.9126
*Centroid *	0.8632	0.9053
*kNN *	0.8478	0.9035
*Winnow *	0.7587	0.8645

**Table 4:** Random sampling rate, token compressing rate, and performances.

*R*_srs_	*R*_tc_	MacroF1	MicroF1
100	100	0.8696	0.9126
90	93	0.8715	0.9136
80	86	0.8677	0.9119
70	79	0.8663	0.9113
60	71	0.8657	0.9114
50	63	0.8653	0.9103
40	53	0.8609	0.9083
30	43	0.8570	0.9051
20	32	0.8517	0.9010
10	19	0.8345	0.8921
